# Organic Acid Production by *Basfia succiniciproducens* from Agro-Industrial By-Products

**DOI:** 10.3390/biotech14030068

**Published:** 2025-09-01

**Authors:** Márta Balázs, Izabella Péter, Hunor Bartos, Zsolt Bodor, Emőke Antal, Csilla Albert, Ildikó Miklóssy

**Affiliations:** 1 Faculty of Science, University of Pecs, Ifjúság 6, H-7624 Pecs, Hungary; 2 Department of Bioengineering, Sapientia Hungarian University of Transylvania, Piata Libertatii, 530104 Miercurea Ciuc, Romania; 3 Research and Development Institute for Wildlife and Mountain Resources, Progresului 35B, 530240 Miercurea Ciuc, Romania

**Keywords:** succinate, organic acid production, *Basfia succiniciproducens*, apple pomace, cheese whey, fermentation

## Abstract

A continuous effort is needed to develop sustainable production methods for industrial platform chemicals. *B. succiniciproducens*, a natural succinic acid-producer, can metabolize five and six carbon atoms containing sugars in pure form as well as from agro-industrial wastes. In our work, we investigate the conversion of industrial by-products, apple pomace from apple juice production, and whey waste from milk processing to succinic acid and other organic acids (lactic, formic, and acetic acid). We obtained a succinic acid yield of 0.224 g/g total consumed fermentable sugars, lactic acid yield was 0.087 g/g, in turn, formic acid was produced at a 0.034 g/g yield, and acetic acid was obtained at 0.010 g/g total consumed fermentable sugars, using a thermal pretreated apple pomace-based medium. In the case of pretreated whey-based medium formulation, we obtained a succinic acid yield of 0.236 g/g consumed lactose, while formic acid and acetic acid were produced as well (0.09 g/g and 0.101 g/g, respectively). We demonstrate that lactose is a promising carbon source for organic acid production by *B. succiniciproducens,* while our study is the first to propose the use of a similarly available agro-industrial by-product, apple pomace, for the fermentative production of succinic acid by *B. succiniciproducens*.

## 1. Introduction

### 1.1. Apple Pomace and Whey as Potential Substrates for Added-Value Compound Production

Apples are one of the most consumed fruits worldwide, with fresh consumption reaching 71%, while 20% of the fruit is processed into various fruit concentrates [1]. In terms of its use as a substrate, due to its fermentable sugar content, apple pomace offers the possibility of biomass and bio-based compound production, like chitosan or xanthan gum [2,3]. A number of successful bioconversion methods of apple pomace have been reported, with several microorganism species (e.g., *Aspergillus niger*, *Rhizopus oryzae*, *Xanthomonas campestris*, *S. cerevisiae*, *Thamnidium elegans*) reported to produce enzymes, aroma compounds, heteropolysaccharides, citric acid, ethanol, or γ-linolenic acid [4].

Another important raw material in food production is bovine milk. Approximately 190–200 million tons of whey are produced annually, and with an estimated growth rate of 1–2%, this amount could reach 200–240 million tons by 2030 [5].

In the cheese-making process, around 80–90% of the milk is leaving the processing as whey, making whey the major by-product of cheese manufacturing [6]. Whey is a yellowish liquid, containing 94% water, and approximately 50% of the dry matter content of milk, the main components being lactose, followed by whey proteins and minerals [1]. Today, 50% of whey is converted and commercialized in the form of fruit yogurt, cheeses, and butter [7,8]. Data from the literature reports a number of products made possible by the bioconversion of lactose or whey, such as bioethanol, antibiotics (nisin), bioplastics, single-cell proteins, and polylactic acid [8,9]. In the production of organic acids, whey can also be a potential carbon source to form succinic acid by, e.g., Actinobacillus *succinogenes*, and as a source of propionic acid produced by *Propionibacterium* [10].

### 1.2. Pretreatment Methods of Agro-Industrial By-Products for Fermentations

In order to obtain fermentable, hydrolyzed sugars (e.g., fructose, glucose, saccharose, galactose, xylose) with increased bioavailability for bacteria, physicochemical pretreatment and enzymatic hydrolysis of complex substrates is necessary, including thermal treatment, pH adjustment, and different incubation times and shaking parameters [11]. Many research efforts aim at combining pretreatment methods and host systems to produce potential bio-based compounds from sustainable sources.

For the pretreatment of fruit and vegetable waste, heat treatment is often used, followed by on-site enzymatic hydrolysis for solid-state fermentations [12]. A similar approach of homogenization followed by thermal pretreatment and an enzyme-cocktail-mediated hydrolysis of soluble sugars was used for succinic acid production by an engineered *Yarrowia lipolytica* strain [13]. There have been studies involving the organic fraction of household kitchen waste as well, which, pretreated by enzymatic hydrolysis, can be a promising substrate for succinic acid production [14].

Chemical pretreatments are mainly used for the lignification of cellulosic materials and are optimized and maximized to this day. In acid pretreatments, concentrated or dilute acid is usually used to dissolve the lignin–hemicellulose matrix. The commonly used acids are H_2_SO_4_, HCl, and HNO_3_ [15], often combined with heat treatments to increase effectiveness [16]. For instance, acetic acid pretreatment followed by enzymatic hydrolysis achieved a cellulose conversion rate of over 98.2%, resulting in a high yield of fermentable sugars [17]. Dilute sulfuric acid treatment optimized at specific temperatures and durations yielded up to 411.4 g of fermentable sugars per kg of dried apple pomace, further fermented to produce bioethanol [18].

The compounds most commonly used in alkaline treatments are NH_4_OH, KOH, NaOH, Ca(OH)_2_, NH_3_, and (NH_4_)_2_SO_3_ [19], which cause structural deformation of lignin, induce swelling of cellulose and thus partial crystallization of cellulose, while partially dissolving hemicellulose, thus facilitate lignocellulosic biomass to be more accessible to enzymatic treatments [20,21]. The combination of heat and acid treatment can maximize the extraction of both pectin and fermentable sugars, making the process more efficient and sustainable [18,21].

Integration of enzymatic treatments with these methods can further enhance sugar release, as enzymes can act on the pretreated biomass to convert polysaccharides into fermentable sugars more effectively, while the choice of enzymes varies according to the main composition of the biomass, and their effects are controlled by the availability of the biomass [22].

While similar treatments significantly enhance the fermentable sugar yield from apple pomace, it is important to consider the potential generation of inhibitors during the process, for example, acetic acid, formic acid, furfural, or 5-hydroxymethylfurfural. Therefore, optimizing treatment conditions to balance sugar yield and inhibitor formation is crucial for efficient industrial applications [23].

### 1.3. Bio-Based Succinic Acid Production

Succinic acid is listed as a platform chemical, and its uses include food, chemical, and pharmaceutical industries as a precursor to many chemicals, such as solvents, perfumes, lacquers, plasticizers, dyes, and photographic chemicals. It also finds application as a surfactant, ion chelator, and as an additive in various industries [24]. Due to its terminal carboxylic acid groups, it opens many avenues to produce other bio-based compounds, such as 1,4-butanediol, which is the starting compound for tetrahydrofuran and other industrially relevant compounds [25].

In terms of microbial metabolism, succinic acid can be produced by three pathways: the oxidative and reductive branches of the tricarboxylic acid cycle and the glyoxylate cycle. Under aerobic conditions, it is formed as an intermediate product of the tricarboxylic acid cycle and ultimately serves as an electron donor. Through biorefinery platforms, succinic acid can be generated through the microbial fermentation of the carbohydrate fraction of several substrates [26]. Microorganisms used for fermentative succinic acid production, even from complex substrates, include *Actinobacillus succinogenes* [27], *Anaerobiospirillum succiniciproducens* [28], *Mannheimia succiniciproducens* [29], and recombinant *Escherichia coli* [30]. Moreover, some of these strains can fix CO_2_ during fermentation and thus reduce greenhouse gas emissions.

There are several sources from the literature that evaluate the potential for the production of succinic acid from diverse types of agro-industrial waste [31]. Data report bio-based production of succinic acid from bread waste [32], corncob using *A. succinogenes* [33], and orange peel by *Fibrobacter succinogenes* [34]. Interestingly, there have been lab-scale experiments for succinic acid production from mixed waste consisting of vegetables, fruits, meat, rice, and noodles in co-cultured fermentations using engineered *E. coli* and *A. succinogenes* [35]. Nowadays, industrial-scale production of succinic acid is possible using metabolically optimized *E. coli* and yeasts, but production from lignocellulosic carbohydrates has not yet been achieved on a commercial scale [36].

As industrial-scale bio-based succinic acid production is still in need of improvements and cost-effective solutions to establish competitive production technologies, both the optimization of metabolic pathways to maximize succinic acid production by metabolic engineering and efforts to investigate more sustainable fermentation processes based on the use of renewable raw materials are in progress [37].

### 1.4. Basfia succiniciproducens—A Novel Microorganism for Succinic Acid Production

*B. succiniciproducens*, a natural succinic acid producer, is a facultative anaerobic bacterium from the Pasteurellaceae family, firstly isolated from bovine rumen [38,39]. Under anaerobic conditions, it can produce significant amounts of succinic acid, reportedly 0.75 mol succinic acid/mol glucose, by utilizing glucose as a carbon source [40]. Metabolic traits, such as using fumarate as a final electron acceptor and its CO_2_ fixation capability, render *B. succiniciproducens* an ideal candidate for the development of new biotechnological industrial routes.

The metabolic pathways and redox balance mechanisms involved in CO_2_ fixation are essential for optimizing succinate yield in *Basfia succiniciproducens*. PEP carboxylase catalyzes the carboxylation of phosphoenol pyruvate to oxaloacetate, incorporating CO_2_ into the metabolic cycle, enhancing the carbon flux towards succinate [37,41]. Studies using ^13^C metabolic flux analysis have shown that CO_2_ fixation via PEP carboxylase is a significant contributor to the overall carbon flux towards succinate [40]. During anaerobic fermentation, *B. succiniciproducens* uses fumarate as a final electron acceptor, which is subsequently reduced to succinate, a process crucial for maintaining redox balance [37]. Malic enzyme and transhydrogenase, in turn, help in maintaining the redox balance by converting NADH to NADPH, which is necessary for anabolic reactions [40].

*B. succiniciproducens* can utilize simple sugars other than glucose, as well as glycerol as a sole carbon source [42]. The use of glycerol allows the formation of fewer by-products and low carbon source requirements, which account for a significant industrial potential [42,43]. It was shown that the *B. succiniciproducens* DD1 strain is capable of metabolizing all types of monosaccharides present in lignocellulose, as well as glycerol [44], with glycerol being found to result in higher g/g yields compared to other carbon sources [44,45].

Applied metabolic engineering strategies for improved succinic acid production in *B. succiniciproducens* include in silico metabolic flux analysis and in vivo ^13^C flux tracking and subsequent strain optimization studies [40,46].

Our previous publications [42,47] investigated the growth dynamics and metabolite production of *B. succiniciproducens* strain DD1 on glucose, glycerol, and xylose carbon sources, respectively. Metabolic flux analysis was used to determine the expected pathways and the production rate of key metabolites, followed by substrate preference studies to investigate biomass production and optical density. The metabolic model (iTY425, [48]) was adjusted to include the anabolic pathway of glycerol, while laboratory-scale fermentation parameters were investigated with special regard to inlet gas composition under simulated anaerobic and microaerobic conditions [47].

Moreover, a handful of publications have investigated succinic acid production by *B. succiniciproducens* starting from industrial non-food resources, reviewed in [37]. Regarding the utilization of lignocellulosic biomass, *B. succiniciproducens* strain BPP7 exhibited co-consumption of the primary sugars, namely glucose and xylose derived from *Arudo donax* hydrolysate, reaching a yield of approximately 0.75 mol/mol and an enhanced productivity of 0.35 g/Lh during pre-pilot fermentations [49]. The study by Salvacha demonstrated that *B. succiniciproducens* CCUG 57335 could grow on corn stover rich in xylose, resulting in a final Y_SA_/C of approximately 0.68–0.69 g/g [36]. A yield of 0.34 g/g of succinic acid was obtained by using lactose concentrate as substrate [50].

The aim of our research is to investigate the use of other agro-industry-derived by-products for the sustainable production of succinic acid in *B. succiniciproducens,* providing initial data for a biotechnological process based on these widely available complex substrates.

The results obtained will allow us insights into a new substrate spectrum of *B. succiniciproducens* using alternative carbon sources, and, to our knowledge, this work is the first to address succinic acid production from apple pomace and liquid cheese whey.

## 2. Materials and Methods

### 2.1. Pretreatments

#### 2.1.1. Apple Pomace, Physico-Chemical Pretreatments

Apple pomace pretreatment steps were carried out based on a modified protocol from [51]. In the first step, 0.5 kg of apple pomace (from a local juice factory) was dried at 68 °C for 36 h, then ground by a laboratory mill (0.2–0.5 mm particle size) and stored at −20 °C until use. Control samples were prepared in 50 mL centrifuge tubes; 5 g of prepared apple pomace was dissolved in 40 mL of distilled water and incubated at room temperature for one hour at 120 RPM, and the resulting sample was labeled as “AC”. In case of heat treatment, a 150 mL centrifuge tube with a mixture of 40 mL distilled water and 5 g apple pomace was used, but, in this case, the treatment was carried out using an autoclave at 121 °C for 15 min. The solids were then separated by centrifugation (13,600 RPM, 10 min, 4 °C). During chemical pretreatments, sulfuric acid and sodium hydroxide (both from Sigma-Aldrich, St. Louis, MO, USA) were used in different concentrations. For the acid pretreatment, 40 mL of 1% and 2% (*v*/*v*%) sulfuric acid solution were added to the solid apple pomace obtained in the previous step, and further heat-treated at 121 °C for 30 min using an autoclave. The alkaline pretreatments were also carried out in the same way, with the difference that sodium hydroxide solution was used at 1% and 2% (*m*/*m*%). During chemical pretreatment, the solids were separated by filtration, followed by repeated washing (5 times) and filtration to remove the chemicals. For filtration, sterile filter paper (15–20 μm pore-sized filter paper, Branchia, Barcelona, Spain) and sterilized distilled water were used, and the process was carried out in a laminar flow box, maintaining sterile conditions. The filtered liquid samples from the first two steps were stored in a refrigerator at −20 °C until the analysis, and the recovered residual apple pomace was also stored under these conditions. For the subsequent enzymatic hydrolysis, Trenolin Rouge, containing pectinase and glucanase from fermentation of *Aspergillus niger*, *Trichoderma reesei,* and *Penicillium funiculosum*, without cinnamoyl esterase activity (Erbslöh, Geisenheim, Germany), was used in a concentration of 0.04 mL/1 L. After the alkaline pretreatment, the separated apple pomace was washed with distilled water and centrifuged again (13,600 RPM, 10 min, 4 °C), and the wet apple pomace was solubilized in 40 mL distilled H_2_O and incubated for 24 h at 50 °C and 120 RPM. At the end of the enzymatic hydrolysis, the solids were separated by centrifugation (13,600 RPM, 10 min, 4 °C) and stored in a refrigerator, keeping both supernatant and pellet. Hot water extraction sample was coded as “W”, acid pretreatment samples were coded as “C1T” and “C2T”, alkaline pretreatment samples were coded as “L1T” and “L2T”, and enzymatic pretreatment samples were coded as “C1R”, “C2R”, “L1R”, and “L2R”.

#### 2.1.2. Whey Pretreatment

Apple pomace pretreatment steps were carried out based on sources from the literature, with adapted protocols carried out as stated below. The control liquid whey sample (untreated whey) was labeled as “SC”. In the first method (S1), 200 mL of whey was heat-treated (90 °C, 20 min) [52]. The precipitated proteins were separated from the liquid phase by centrifugation (18,000 RPM, 10 min, 6 °C), followed by filtration with a sterile 0.45 µm filter (Carl Roth, Karlsruhe, Germany). The second treatment (S2) was a modified version of the first method, where only the temperature of heat treatment was changed (121 °C, 20 min). For the third method (S3) based on [53], 0.23 g of MgSO_4_·7H_2_O (Carl Roth)was added to 200 mL of whey, followed by a protein precipitation. For method S4 [54], pH was adjusted to 7 using 1 M sodium hydroxide, followed by a heat extraction (70 °C, 10 min), followed by centrifugation (18,000 RPM, 10 min, 6 °C). For the S5 method based on [55], centrifugation (18,000 RPM, 10 min, 6 °C) was followed by vacuum filtration (15–20 μm pore-sized filter paper, Branchia), then protein precipitation (121 °C, 20 min), and then centrifugation (18,000 RPM, 10 min, 6 °C). The prepared samples were kept at −20 °C until analysis. All pretreatment experiments were performed in duplicates.

### 2.2. Medium Composition Optimization in Small-Volume Fermentations

#### Strain

*B. succiniciproducens* wild type (DSM-22022) isolated from cow rumen was purchased from Leibniz Institute DSMZ-German Collection of Microorganisms and Cell Cultures. The cells came in freeze-dried form and rehydration was carried out in a specified TSB medium, containing 17 g of peptone from casein L^−1^, 3 g of peptone from soymeal L^−1^, 2.5 g of glucose L^−1^, 5 g of NaCl L^−1^, 2.5 g of K_2_HPO_4_ L^−1^, and a pH of 7.0. As a first step in our experiment, we performed small-volume tests in duplicates. Cells stored at −80 °C were resuspended in TSB medium (Casein hydrolyzate–peptone (Merck, Darmstadt, Germany), 17 g/L, soy hydrolyzate–peptone (VWR, Radnor, PA, USA), 3 g/L, NaCl (Sigma-Aldrich), 5 g/L, K_2_HPO_4_ (Merck), 2.5 g/L, glucose (Sigma-Aldrich), 20 g/L), then further inoculated in 100 mL of TSB medium with an initial optical density of 0.3, incubated at 130 RPM, and 37 °C for 12 h. Cells were then separated by centrifugation (3500 RPM, 22 °C, 10 min) and resuspended in 5 mL of substrate-free 1×M9 medium. Components of M9 macroelement stock solution at 5× concentration: 17.7 g/L of Na_2_HPO_4_ (Chempur, Piekary Śląskie, Poland), 17 g/L of KH_2_PO_4_ (Merck), 10 g/L of NH_4_Cl (Chimopar, Bucharest, Romania), and 2.5 g/L of NaCl (Sigma-Aldrich). The components were dissolved in distilled water for both nutrient solutions and sterilized in an autoclave (MLS3020U, Panasonic, Osaka, Japan) at 121 °C for 30 min. The M9 culture medium contains trace elements, and the components of the 1000× trace element stock solution are 50 mM of FeCl_3_·6H_2_O (Fluka, Charlotte, NC, USA), 20 mM of CaCl_2_ (Chimopar), 10 mM of MnCl_2_·4H_2_O (VWR), 10 mM of ZnSO_4_·7H_2_O (Sigma-Aldrich), 2 mM of CoCl_2_ (Chimreactiv, Bucharest, Romania), 2 mM of CuCl_2_·2H_2_O (Sigma-Aldrich), 2 mM of NiCl_2_·6H_2_O (Merck), 2 mM of Na_2_MoO_4_·2H_2_O (Merck), 2 mM of Na_2_SeO_3_·5H_2_O (Sigma-Aldrich), 2 mM of H_3_BO_3_ ·5H_2_O (Sigma-Aldrich), and 60 mM of HCl (Merck). The following heat-sensitive microelements were added to the 1× dilution of the previously described 5× M9 stock solution at the beginning of the fermentation: 1× trace element solution, 0.02 mM/L of CaCl_2_ (Chimopar), 2 mM/L of MgSO_4_ (VWR), and 2 g/L of fructose or lactose as a carbon source. The resulting cells were inoculated into two 50 mL of 5 g/L of fructose- or lactose-containing 1×M9 media and placed again in a shaking incubator at 37 °C for 12 h. The resulting cells were again separated by centrifugation (3500 RPM, 22 °C, 10 min) and resuspended in substrate-free 1×M9 medium, also with an initial optical density of 0.3. The composition of the prepared media is summarized in Table 1.

Finally, 300 µL of each sample, together with cell-free control samples, was loaded onto the microtitration plate (96-well pureGrade microtitration plate, BRANDplates, Wertheim, Germany), incubated and monitored in a microplate spectrophotometer (FLUOstar OPTIMA, BMG Labtech, Ortenberg, Germany) and run with the detection settings: 600 nm wavelength, 200 cycles, 360 s cycle time, 10 flashes per well, 60 s shaking time before each cycle, and a temperature of 37 °C. Optical density measured at 600 nm was used to calculate specific growth rate in the exponential phase (0–3 h).

### 2.3. Bioreactor Fermentations

The experiments were carried out in a 1 L BIOSTAT A Plus, Sartorius bioreactor. An amount of 0.5 L of the prepared medium containing substrate in 1×M9 (Table 2) was sterilized together with the reactor and inoculated after cooling, starting from an OD_600_ = 0.3. Cultures were monitored for 12 h with the following parameters: 37 °C, 130 RPM mixing (one piece Rushton impeller), pH of 7, air flow of 60 L/min of CO_2_, and 60 L/min of air. Samples were taken every two hours for spectrophotometric measurements, and 1.5 mL samples were centrifuged at 14,000 RPM and stored at −20 °C until analytical measurements.

### 2.4. Analytical Procedures

Samples from reactor fermentation culture supernatants were filtered through a 0.45 µm sterile filter and placed in a sterile HPLC vial at a 50-fold dilution. Equimolar reference solutions were prepared from 20 g/L of D(+)-sucrose (Carl Roth), 20 g/L of D(+)-glucose (Sigma-Aldrich), 20 g/L of D(−)-fructose (Sigma-Aldrich), 20 g/L of lactose monohydrate (Carl Roth), 50 mM of acetic acid (Sigma-Aldrich), and 50 mM of succinic acid (Sigma-Aldrich), 50 mM lactic acid (Sigma-Aldrich), and 50 mM of formic acid (Chimopar) solutions and 5-point standard curves were obtained for proper concentration analysis (see the Supplementary File, Figures S1–S5 and Table S1).

Measurements were performed using a 1260 Infinity liquid chromatograph (Agilent Technologies, Santa Clara, CA, USA) equipped with an isocratic pump, Coregel H3 column (30 cm), fluorescence detector, diode array detector (organic acid detection), and refractive index detector (carbohydrate detection). Detection was performed at a wavelength of 210 nm with an injection volume of 20 µL at a flow rate of 0.6 mL/min, the column temperature was 50 °C, and the mobile phase was a 0.008 N sulfuric acid (Sigma-Aldrich) solution. Raw chromatography data were analyzed and visualized by ChemStation software Rev. C.01.06 (Agilent).

### 2.5. Statistical Analysis

Data processing and visualization were carried out using Microsoft Excel 2021, (Microsoft Corporation, Redmond, WA, USA) and Adobe Illustrator 2021 (Adobe, San Jose, CA, USA).

## 3. Results and Discussion

### 3.1. Effect of Pretreatment Methods on Carbohydrate Content of Apple Pomace and Whey

**Figure 1:**
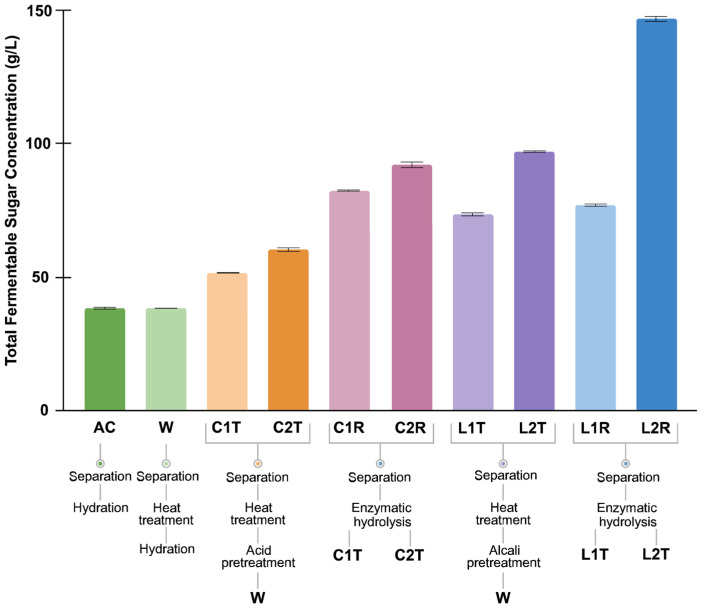
Fermentable sugar content (glucose, fructose, sucrose) of apple pomace following combined extraction methods. AC—control sample, W—hot water extraction, C1T—hot water extraction followed by acid hydrolysis with 1% H_2_SO_4_. C2T—hot water extraction followed by acid hydrolysis with 2% H_2_SO_4_, C1R—hot water extraction followed by 1% H_2_SO_4_ + pectinase hydrolysis, C2R—hot water extraction followed by 2% H_2_SO_4_ + pectinase hydrolysis, L1T—hot water extraction followed by alkaline hydrolysis with 1% NaOH. L2T—hot water extraction followed by alkaline hydrolysis with 2% NaOH, L1R—hot water extraction followed by 1% NaOH + pectinase hydrolysis, L2R—hot water extraction followed by 2% NaOH + pectinase hydrolysis. The error bars represent the SDs based on duplicate experiments.

Chemical and enzymatic pretreatment of complex materials derived from industrial sources is necessary to ensure proper availability of fermentable carbon sources for downstream microbial processes. Here, we present an investigation of a wide array of pretreatment methods, both physico-chemical and enzymatic, with regard to obtaining the highest concentration of fermentable sugars for *B. succiniciproducens* biomass production and succinic acid formation. Pretreated apple pomace and whey, achieving high fermentable sugar yield, were investigated for biomass production; thus, potential industrial applicability during small-scale fermentation tests, followed by fed-batch bioreactor fermentation experiments for the investigation of process parameters. In the case of apple pomace, in order to obtain fermentable sugars that can be taken up by *B. succiniciproducens*, physico-chemical pretreatment and enzymatic hydrolysis are necessary. These steps are important for the improvement of raw material utilization, succinic acid production, and the reduction in by-product formation. Apple pomace pretreatment methods were elaborated based on data from the literature [51] and comprising a series of physico-chemical treatments, such as hot water extraction or alkaline hydrolysis and enzymatic conversion of the polysaccharide fraction of apple pomace derived from a regional small fruit processing plant. A combination of these extraction methods was used to enhance the formation of simple sugars and to determine if there were significant differences in fermentable sugar content between pretreatment strategies. The differences in the concentrations of fermentable sugars (sucrose, glucose, and fructose) following different physico-chemical and enzymatic pretreatments are represented in Figure 1. Heat treatment alone resulted in a 38.3 g/L fermentable sugar content (sucrose, glucose, and fructose), while both acidic and alkali pretreatments increased bioavailable sugar formation (51.6 g/L—1% acid, 60.2 g/L—2% acid, 73.3 g/L—1% alkali, and 96.8 g/L—2% alkali, respectively). The process was further enhanced by enzymatic hydrolysis, the highest fermentable sugar concentration being achieved in the case of heat extraction, 2% alkaline hydrolysis, and enzymatic treatment, resulting in a total fermentable sugar concentration of 147.0 g/L.

Our results are comparable with other relevant data from the literature: dilute H_2_SO_4_ pretreatment at 91 °C for 16 min resulted in a glucose yield of 13.9 g/100 g DW [56]; moreover, in a similar study, pretreatment with 2% H_2_SO_4_ resulted in a 44 g/L concentration of total fermentable sugars, while the utilization of 2% NaOH led to the formation of 19.1 g/L fermentable sugars, respectively [57]. Interestingly, in a recent study, acidic pretreatment with a lower concentration of sulfuric acid (0.5%) gave higher sugar yield (69.6 g/L fermentable sugars) when compared to 1.5% sulfuric acid pretreatment (67.3 g/L fermentable sugars [58]), while, in our case, 2% H_2_SO_4_ treatment with thermal extraction was more efficient, resulting in 60.18 g/L total sugar formation.

Regarding the simple sugar composition of pretreated apple pomace presented in the Supplementary File, Figure S6, H_2_SO_4_ pretreatment yielded the highest fructose concentrations among the tested methods (32.44, 41.01, 46.32, and 61.64 g/L), whereas NaOH pretreatment favored sucrose hydrolysis (38.13, 61.7, 38.13, and 104.91 g/L). Considerable glucose formation was observed only after H_2_SO_4_ pretreatment and pectinase hydrolysis (21.95 and 23.36 g/L).

Our results indicate that the combination of methods such as hot water extraction, acid and alkaline treatment, and enzymatic hydrolysis does result in higher carbohydrate concentrations (more than 3.8× compared to heat treatment alone). However, we considered the complexity of the combined process not to be the most advantageous in terms of laboratory-scale applications.

Apple pomace prepared with heat treatment was further studied as media or an additive in fermentations with *B. succiniciproducens*.

**Figure 2:**
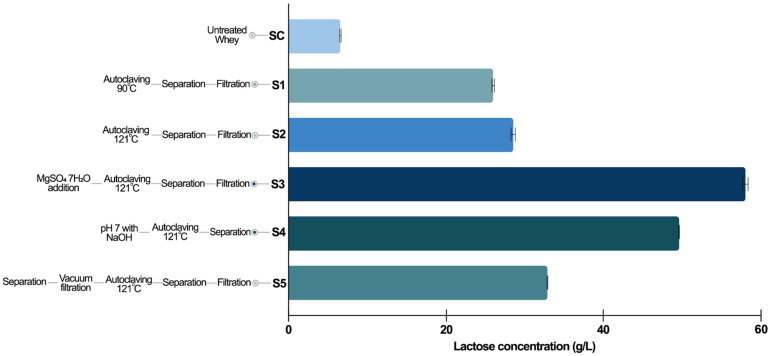
Lactose concentration of whey following different extraction methods. Schematic flowchart of pretreatment methods applied to whey. SC—control sample. S1—protein precipitation at 90 °C 20’ and thermal extraction. S2—protein precipitation at 121 °C 20’ and thermal extraction. S3—MgSO_4_·7H_2_O treatment, and thermal extraction. S4—pH 7 NaOH, autoclaving, separation. S5—centrifugation at 18,000 RPM 10’ 6 °C, vacuum filtration, autoclaving, separation, and filtration. The error bars represent the SDs based on duplicate experiments.

The effects on lactose content of whey subjected to different physico-chemical treatments are represented in Figure 2. According to our results, lactose concentration in the untreated whey (Figure 2) was 12.0 g/L, which increased significantly to 57.8 g/L following the most successful pretreatment method, carried out by the addition of MgSO_4_·7H_2_O (S3, Figure 2). In parallel, the fourth (S4, Figure 2) and fifth (S5, Figure 2) treatments resulted in an increase in lactose concentration to 49.4 g/L and 32.8 g/L, respectively. Our results are similar to other data presented in the literature, where a concentration of 50 ± 2 g/L lactose was recorded from yogurt after thermal coagulation, separation, and 1N HCl acid treatment, followed by filtration and autoclaving, while rehydrated whey powder resulted in 210 g/L lactose after MgSO_4_·7H_2_O addition, autoclaving, and centrifugation [53,59]. It is known that different whey pretreatment methods can increase lactose concentration due to volume reduction, protein precipitation, and freeing of bound lactose from albumins or other proteins [60].

Taking into account a potential industrial application of these pretreatment processes, in the case of whey, the S4 (higher speed centrifugation, vacuum filtration) has been further tested in fermentation media optimization experiments.

### 3.2. Medium Composition Optimization in Small-Volume Fermentations

To assess the applicability of our pretreated industrial by-products as fermentation substrates, microplate volume fermentations were set up, and several experiments were carried out with different medium compositions. We were also curious about the population dynamics of *B. succiniciproducens* if the pretreated apple pomace or whey was used as the sole substrate, in equal volume with M9 mineral medium, or if the pretreated substrates were supplemented with the trace element mixture of the M9 medium. We monitored culture-specific growth rates in comparison to fermentations with fructose and lactose-containing M9, which were used as reference cultures. It is noteworthy that the use of fructose or lactose as carbon sources for *B. succiniciproducens* has not been studied to this extent in previous works.

**Figure 3:**
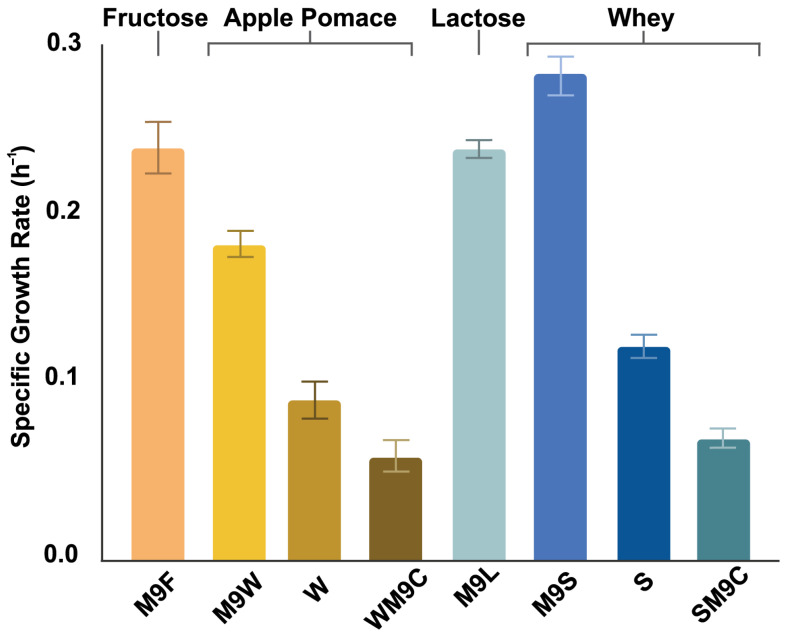
Specific growth rates of microplate volume fermentations of *B. succiniciproducens* grown on different media. M9F, M9L: M9 minimal medium with fructose or lactose as C-source. M9W: autoclave-centrifugation-treated apple pomace in a 1:1 ratio with M9. W: autoclave-centrifugation-treated apple pomace. WM9C: apple pomace supplemented with the trace minerals from the M9. M9S: pH7 and thermal extraction-treated whey in a 1:1 ratio with M9. S: pretreated whey used as sole medium. SM9C: treated whey supplemented with M9 trace minerals. Samples were measured in duplicates, and error bars represent standard deviation values.

Specific growth rates of *B. succiniciproducens* cultures grown on different media are illustrated in Figure 3, from which the reference culture, fructose minimal broth (M9F), had the highest specific growth rate of 0.255 h^−1^, regarding apple pomace. However, the medium denoted M9W, which contained autoclave-centrifugation-treated apple pomace in a 1:1 ratio with M9 minimal medium, registered a specific growth rate of 0.195 h^−1^, close to the reference culture. In the case of whey, the medium consisting of equal parts M9 medium and substrate obtained by the fourth pretreatment method seems to be the best for our strain, as the specific growth rate was the highest in this case (0.301 h^−1^, see Figure 3); thus, whey adequately supports the activity of *B. succiniciproducens*.

### 3.3. Fermentation Profile Analysis of B. succiniciproducens Cultures Grown on Pretreated Apple Pomace

**Figure 4:**
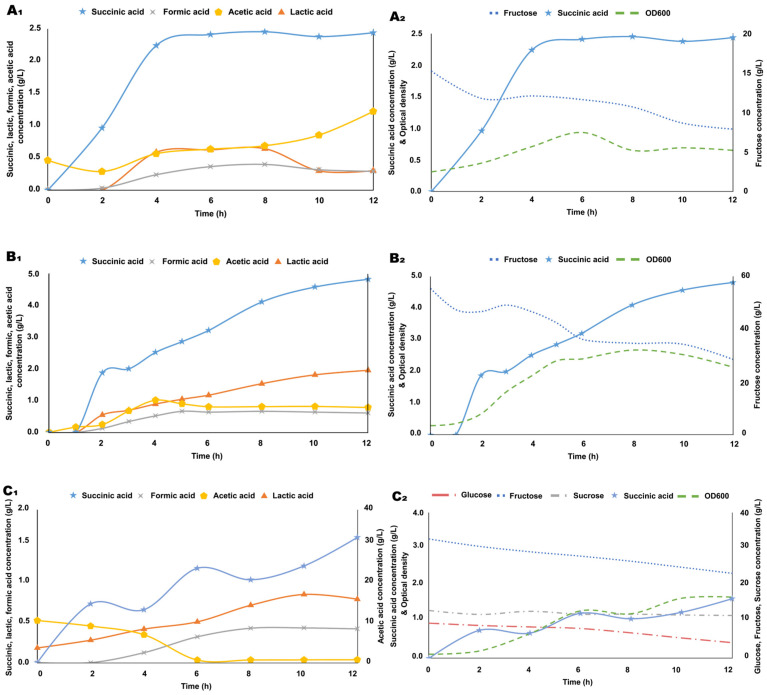
Substrate consumption and organic acid production of *B. succiniciproducens* cultures. Bioreactor fermentation profiles of 15 g/L fructose (**A_1_**,**A_2_**), 55 g/L fructose (**B_1_**,**B_2_**), and pretreated apple pomace (**C_1_**,**C_2_**) substrate-containing cultures. Fructose, glucose, sucrose, lactic acid, formic acid, acetic acid, and succinic acid concentrations and OD_600_ values are represented in the time course of fermentations.

Pretreated apple pomace was further analyzed by performing bioreactor batch fermentations in *B. succiniciproducens* cultures grown in treated apple pomace-supplemented media vs. cultures grown in medium containing fructose as a sole carbon source, as a reference culture. During fermentations, changes in the specific growth rate, substrate consumption, and metabolite production were monitored (Figure 4).

Bioreactor fermentation using an apple pomace-derived substrate was performed in parallel with control cultures grown on fructose as the sole substrate (Figure 4(A_2_,B_2_)). We observed a substrate consumption rate of 0.61 g/Lh at a lower starting concentration of fructose and a 2.24 g/Lh utilization rate at an initial fructose concentration of 55 g/L. In the case of the medium based on apple pomace pretreated with thermal extraction (autoclaving), fructose, glucose, and sucrose concentrations were monitored, among which fructose seems to be the best utilized carbon source (Figure 4(C_2_)), while all three sugars are consumed in a near-linear fashion in this experimental setup. However, we observed an auxotrophic-like culture dynamics on this substrate, which seems to be correlated with acetic acid concentration, which, to our surprise, seems to function as a substrate for *B. succiniciproducens* under these conditions: 11.008 g/L acetic acid at the start of the fermentation, 0.68 g/L at 6 h of fermentation, correlated with an inflection point in the growth curve, and 0.778 g/L at the end of the experiment, reaching a consumption rate of 1.72 g/Lh at half of the fermentation (Figure 4(C_1_,C_2_)). Our succinic acid yield from these experiments is similar to the succinic acid yield of 0.22 g/g formerly achieved using *Arundo donax* hydrolysate as a substrate [61].

This surprising finding could explain the adaptability of *B. succiniciproducens* to relatively high acetic acid concentrations observed by [49] in *Arudo donax* hydrolysate-grown cultures. Nevertheless, the use of acetic acid as a potential carbon source for fermentation is gaining interest, and it has been shown to be utilized either as a sole carbon source or as a co-substrate in several engineered or wild-type microbial strains, e.g., *E. coli*, *Corynebacterium glutamicum*, *Pseudomonas putida*, *Saccharomyces cerevisiae*, *Cryptococcus curvatus*, *Rhodotorula glutinis*, *Yarrowia lipolytica*, and *Aspergillus oryzae* for the production of acids, alcohols, esters, and other chemicals [62].

**Figure 5:**
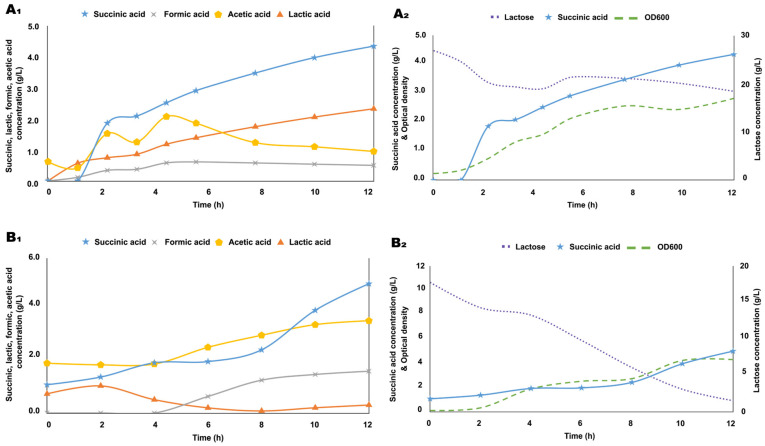
Substrate consumption and organic acid production of *B. succiniciproducens* grown on a whey-based substrate. Bioreactor fermentation profiles of the control culture grown on 27 g/L of lactose (**A_1_**,**A_2_**) and pretreated whey-based medium (**B_1_**,**B_2_**). Lactose, lactic acid, formic acid, acetic acid, and succinic acid concentrations and OD_600_ values are represented in the time course of fermentations.

To investigate the succinic acid production potential of the whey-based medium in a bioreactor setting, first, we performed a control fermentation with 26.77 g/L of lactose as the sole carbon source, which was consumed at a rate of 0.7 g/Lh during the first 12 h of fermentation and yielded 4.33 g/L of succinic acid (Figure 5(A_1_,A_2_)). On the other hand, the whey-based medium proved to be superior in terms of substrate consumption, maximal biomass, and succinic acid yield as well, reaching 0.61 g/Lh of substrate utilization rate (lactose), and a 5.00 g/L of succinic acid concentration along with a robust specific growth rate of 0.31 h^−1^ (Figure 5(B_1_,B_2_)). Lactic acid was the primary organic acid produced in the lactose-based fermentation, while in the whey-based medium, there is a significant lactic acid concentration present in the initial composition as well.

### 3.4. Comparison of the Metabolic Potential of B. succiniciproducens Grown on Pretreated Apple Pomace and Cheese Whey

According to our results, lactose is a promising carbon source for succinic acid production by *B. succiniciproducens*, as lactose-containing mediums resulted in the highest succinic acid yield in our experimental setting, namely: 0.276 g of succinic acid/g of lactose in the case of the 27 g/L of lactose control medium, while the whey-based medium had a similar yield of 0.236 g/g (Table 3, effect-size comparisons are presented in Table S2).

Although it is known that *Basfia* strains are able to utilize a wide array of carbon sources, including lactose [38,47], lactose-containing industrial by-products have not been investigated until recently for the production of succinic acid by this species [50]. Our results with the whey-based medium are similar to the results of Terboven [38,50,62], who obtained a succinic acid yield in a similar medium based on lactose concentrate of 0.34 g/g and a higher, 0.67 g/g of succinic acid/lactose yield in the lactose concentrate supplemented with yeast extract. Nevertheless, the medium formulation used in our study can be considered a more basic, straightforward approach, with a possibility of direct use without the costly concentration technology used for whey permeate and lactose concentrate; thus, it might be more advantageous in terms of industrial applications.

Finally, to our knowledge, our study is the first to propose the use of a similarly available agro-industrial by-product, apple pomace, for the fermentative production of succinic acid by *B. succiniciproducens*. Our results show that, compared to control fermentations conducted with fructose as a sole carbon source, thermal extraction prepared apple pomace yielded a similar succinic acid production (0.224 g/g) as the medium containing 15 g/L of fructose (0.141 g/L), while high fructose concentration had a negative effect on succinic acid production, with a modest yield of 0.072 g/g fructose. However, the succinic acid yield compared to the initial agro-industrial by-product quantity favors apple pomace (0.033 g of succinic acid/g raw apple pomace versus 0.01 g of succinic acid/g of liquid cheese whey).

## 4. Conclusions

Complex materials from agro-industrial processing by-products contain a significant fraction of macro- and micronutrients, namely carbohydrates, proteins, and minerals, which can be further processed by microbial fermentation into bio-based added-value compounds.

In our study, we demonstrate the application of simple pretreatment methods for increased availability of fermentable sugars from apple pomace and liquid whey, based on thermal treatment/thermal treatment and desalting, respectively. We demonstrate the direct use of liquid whey in fermentation media for *B. succiniciproducens* without the costly concentration technology used for whey permeate and lactose concentrate. Both substrates, used in combination with chemically defined mineral growth media, ensured high specific growth rates (in the case of whey, an exceptionally high specific growth rate of 0.301 h^−1^) for *B. succiniciproducens*; thus, our results support the industrial applicability of these substrates. Using apple pomace as a fermentative substrate in a bioreactor, with a specific growth rate of 0.152 h^−1^, the succinic acid yield achieved 0.224 g/g. However, a large amount of lactic acid was produced as the main by-product, with a yield of 0.087 g/g, while the amounts of formic and acetic acid were significantly lower.

In the case of whey, the specific growth rate is outstanding in a bioreactor setting as well, 0.310 h^−1^, along with a succinic acid yield of 0.23 g/g. Lactic acid appears in large quantities in the fermentation broth at the beginning of the fermentation, and production of acetic and formic acid is significant, 0.101 g/g and 0.100 g/g, respectively. The co-production of these organic acids creates downstream separation challenges that might influence economic viability; thus, strain optimization by metabolic engineering seems critical to ensure the viability of biotechnologies addressing succinic acid production from cheese whey by this strain.

No bibliographic data was found to compare succinic acid yield from liquid whey in *B. succiniciproducens*; consequently, our results sustaining industrial applicability of this valorous substrate in *B. succiniciproducens* fermentations represent scientific novelty in *Basfia* biotechnology. The starting raw materials and the medium formulations used in our study can be considered a more straightforward approach, an approach that proves to be of utmost importance considering the growing pressure for developing economically feasible, sustainable processes.

## Figures and Tables

**Table 1:** Composition of culture media used for small-volume medium optimization experiments.

Code	Composition	Code	Composition
M9L	M9 + 5 g/L lactose	M9F	M9 + 5 g/L fructose
M9S	M9 + S (1:1)	M9W	M9 + W (1:1)
S	S	W	W
SM9C	S + M9 micronutrients	WM9C	W + M9 micronutrients

**Table 2:** Medium formulations for bioreactor cultures of *Basfia succiniciproducens*.

Medium	Substrate	Concentration
1×M9	Fructose	15 g/L
	Fructose	55 g/L
	Lactose	27 g/L
	Pretreated apple pomace: 1×M9	1:1
	Pretreated liquid whey: 1×M9	1:1

**Table 3:** Comparison of the concentrations and yields of succinic acid and other organic acids produced by *B. succiniciproducens* in fermentation medium containing different carbon sources.

	15 g/L Fructose	55 g/L Fructose	27 g/L Lactose	Apple Pomace (W) *	Whey (S)
Substrate consumption (g/Lh)	0.610	2.244	0.700	0.610	1.383
Specific growth rate (h^−1^)	0.183	0.421	0.308	0.152	0.310
Succinic acid concentration (g/L)	2.433	4.811	4.334	1.637	5.007
Formic acid concentration (g/L)	0.292	0.610	0.500	0.443	1.619
Acetic acid concentration (g/L)	1.217	0.779	0.950	0.779	3.573
Lactic acid concentration (g/L)	0.301	1.947	2.319	0.829	0.468
Succinic acid yield (g/g consumed sugar)	0.141	0.072	0.276	0.224	0.236
Overall yield (g succinic acid/g agro-industrial byproduct)				0.038	0.008

* Substrate consumption rates determined from total concentrations of fructose, glucose, and sucrose, similar to other works [61].

## Data Availability

The original contributions presented in this study are included in the article/Supplementary Material. Further inquiries can be directed to the corresponding author(s).

## Supplementary Materials

The following supporting information can be downloaded at: https://www.mdpi.com/article/10.3390/biotech14030068/s1, Figure S1: HPLC chromatograms of fermentation sample taken at the 12th hour of bioreactor fermentation on pretreated apple pomace (Raw chromatogram obtained with ChemStation software, Agilent); Table S1: Retention times of organic acids and sugars (ChemStation, Agilent; Figure S2: 5 point calibration curve for acetic acid (ChemStation, Agilent); Figure S3: Calibration curve for fructose (ChemStation, Agilent); Figure S4: Calibration curve of glucose (ChemStation, Agilent); Figure S5: Calibration curve for Succinic Acid (ChemStation, Agilent); Figure S6: Fermentable sugar composition (glucose, fructose, sucrose) of apple pomace following combined extraction; Table S2: Percent change in fermentation parameters for *B. succiniciproducens* grown on different carbon sources, expressed relative to the values obtained with 15 g/L fructose as the baseline. Positive values indicate an increase, and negative values indicate a decrease compared to the baseline. Values are derived from single bioreactor experiments for each carbon source.
