# Supplementary material for: Organic Acid Production by Basfia succiniciproducens from Agro-Industrial By-Products
Source: BioTech (Basel). 2025 Sep 1;14(3):68. doi: 10.3390/biotech14030068 (PMC12452410; doi:10.3390/biotech14030068)
Supplement: Supplementary file 1 [file biotech-14-00068-s001.zip › biotech-3796306-supplementary.pdf]

BioTech

Article

Organic Acid Production by *Basfia succiniciproducens* from Agro-Industrial By-Products

Márta Balázs [1], Izabella Péter [2], Hunor Bartos [2,3], Zsolt Bodor [2,3], Emőke Antal [1], Csilla Albert [2] and Ildikó Miklóssy [1,2,*]

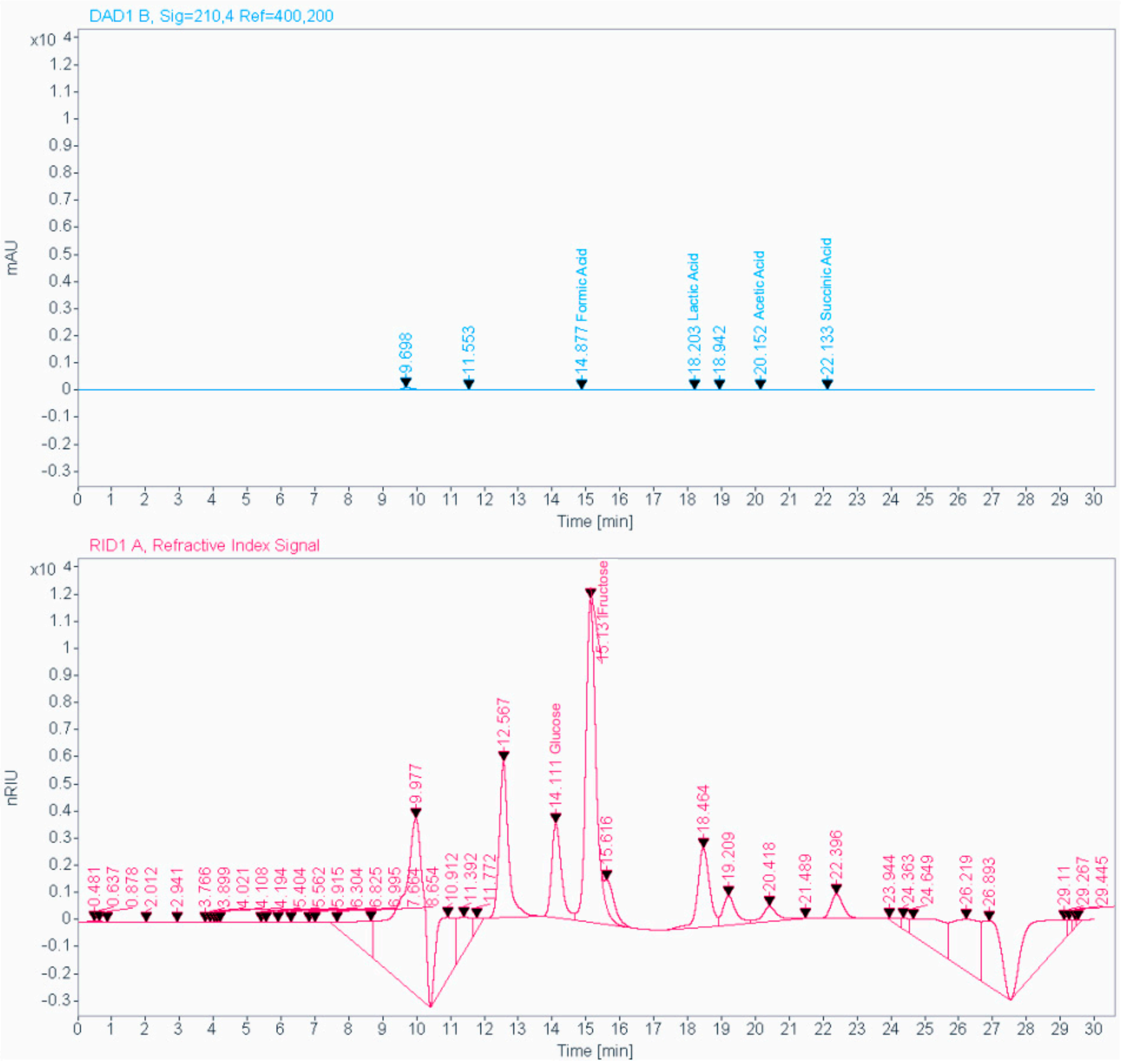

Figure S1. HPLC chromatograms of fermentation sample taken at the 12th hour of bioreactor fermentation on pretreated apple pomace (Raw chromatogram obtained with ChemStation software, Agilent)

Table S1. Retention times of organic acids and sugars (ChemStation, Agilent).

Percent report based on Area

Name	RT [min]	Area	Height	RF	Amount [ng/ul]
Glucose	14.111	69751.9141	3483.0608	0.00000	0.225
Formic Acid	14.877	96.0380	3.7245	0.03040	2.920
Fructose	15.131	255289.0313	11941.2588	0.00000	0.817
Lactic Acid	18.203	340.1419	15.4732	0.00542	1.843
Acetic Acid	20.152	190.7130	8.4849	0.02163	4.126
Succinic Acid	22.133	190.4734	7.1465	0.01910	3.638

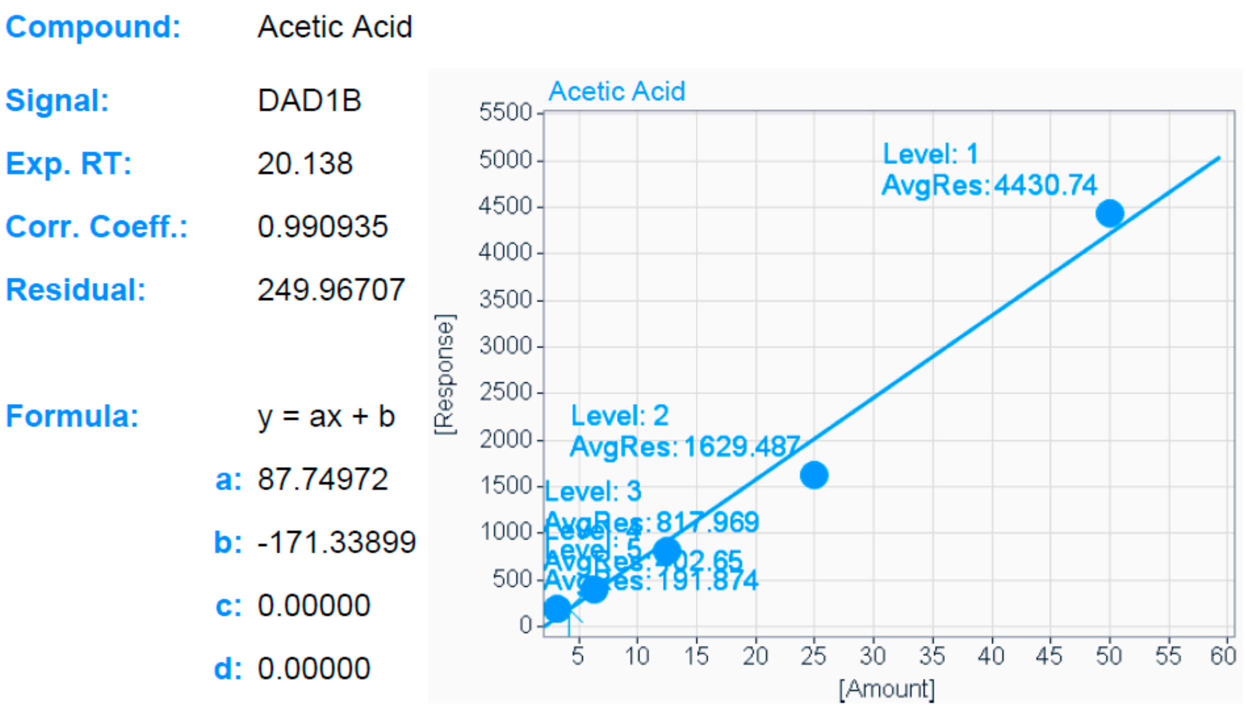

Figure S2. 5 point calibration curve for acetic acid (ChemStation, Agilent).

Compound: Fructose

Signal: RID1A

Exp. RT: 15.113

Corr. Coeff.: 0.999988

Residual: 13447.92637

Formula: y = ax + b

a: 324925.27562

b: -10262.20714

c: 0.00000

d: 0.00000

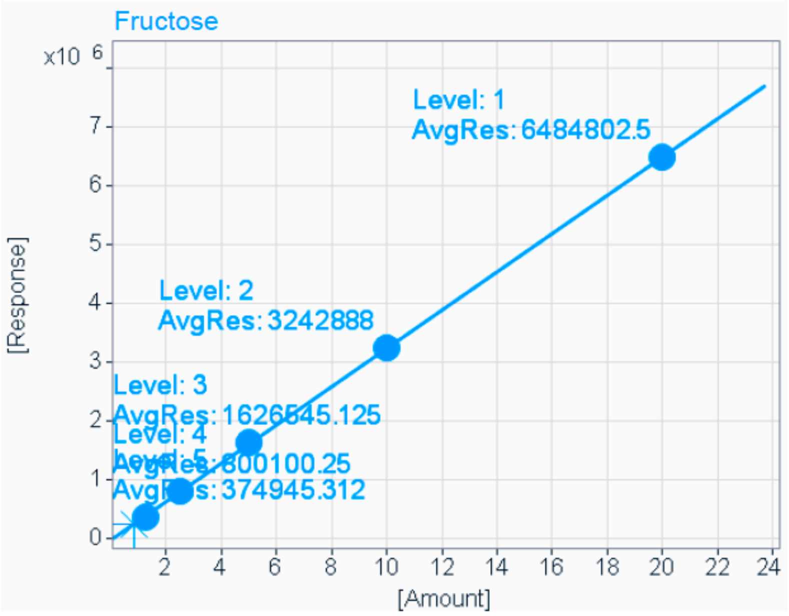

Figure S3. Calibration curve for fructose (ChemStation, Agilent).

Compound: Glucose

Signal: RID1A

Exp. RT: 14.097

Corr. Coeff.: 0.999996

Residual: 7221.01336

Formula: y = ax + b

a: 310587.38366

b: -65.32679

c: 0.00000

d: 0.00000

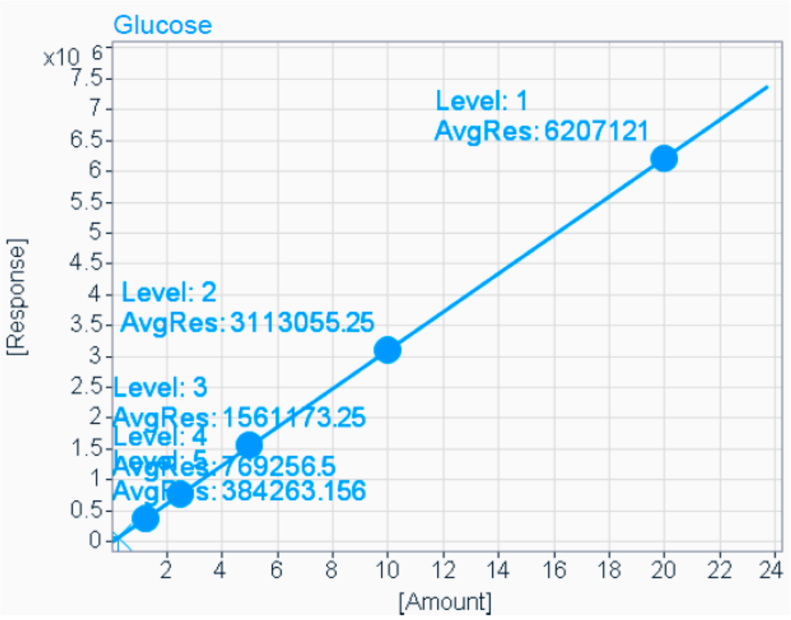

Figure S4. Calibration curve of glucose (ChemStation, Agilent).

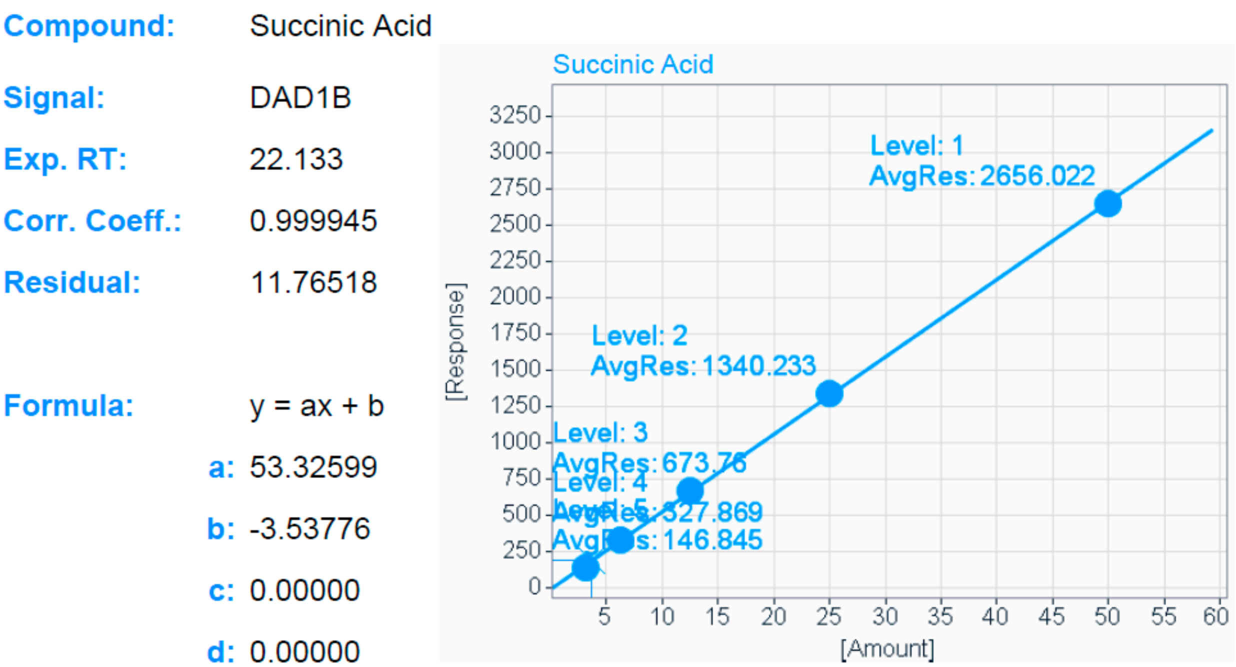

Figure S5. Calibration curve for Succinic Acid (ChemStation, Agilent).

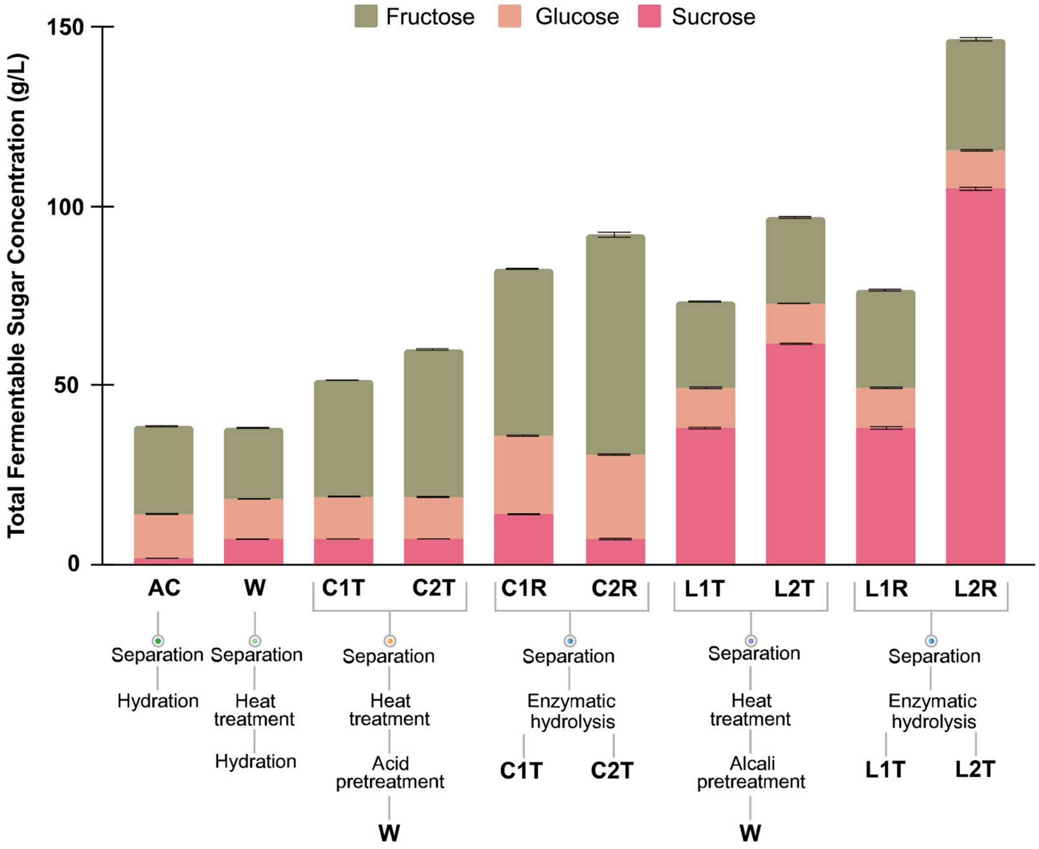

Figure S6. Fermentable sugar composition (glucose, fructose, sucrose) of apple pomace following combined extraction.

Table S2. Percent change in fermentation parameters for *B. succiniciproducens* grown on different carbon sources, expressed relative to the values obtained with 15 g/L fructose as the baseline. Positive values indicate an increase, and negative values indicate a decrease compared to the baseline. Values are derived from single bioreactor experiments for each carbon source.

Carbon Source.	15 g/L fructose	55 g/L fructose	27 g/L lactose	Apple pomace (W)*	Whey (S)
Substrate consumption (g/Lh)	0.0	267.87	14.75	0.0	126.72
Specific growth rate (h-1)	0.0	130.05	68.31	-16.94	69.4
Succinic acid concentration (g/L)	0.0	97.74	78.13	-32.72	105.8
Formic acid concentration (g/L)	0.0	108.9	71.23	51.71	454.45
Acetic acid concentration (g/L)	0.0	-35.99	-21.94	-35.99	193.59
Lactic acid concentration (g/L)	0.0	546.84	670.43	175.42	55.48
Succinic acid yield (g/g consumed sugar)	0.0	-48.94	95.74	58.87	67.38

.
